# Establishment of a Human iPSC Line from Mucolipidosis Type II That Expresses the Key Markers of the Disease

**DOI:** 10.3390/ijms26083871

**Published:** 2025-04-19

**Authors:** Maria Eduarda Moutinho, Mariana Gonçalves, Ana Joana Duarte, Marisa Encarnação, Maria Francisca Coutinho, Liliana Matos, Juliana Inês Santos, Diogo Ribeiro, Olga Amaral, Paulo Gaspar, Sandra Alves, Luciana Vaz Moreira

**Affiliations:** 1 Centre for the Study of Animal Science (CECA)-Institute of Sciences, Technologies and Agroenvironment (ICETA), University of Porto, Praça Gomes Teixeira, Apartado 55142, 4051-401 Porto, Portugal; up201906113@up.pt (M.E.M.); mariana.goncalves@insa.min-saude.pt (M.G.); ana.duarte@insa.min-saude.pt (A.J.D.); marisa.encarnacao@insa.min-saude.pt (M.E.); francisca.coutinho@insa.min-saude.pt (M.F.C.); liliana.matos@insa.min-saude.pt (L.M.); juliana.santos@insa.min-saude.pt (J.I.S.); diogo.ribeiro@insa.min-saude.pt (D.R.); olga.amaral@insa.min-saude.pt (O.A.); 2 Research and Development Unit, Department of Human Genetics, National Institute of Health Doutor Ricardo Jorge, INSA I.P., Rua Alexandre Herculano, 321, 4000-055 Porto, Portugal; 3 Biology Department, Faculty of Sciences, University of Porto, Rua do Campo Alegre, 4169-007 Porto, Portugal; 4 Associate Laboratory for Animal and Veterinary Sciences, AL4AnimalS, Faculty of Veterinary Medicine, University of Lisboa, Avenida da Universidade Técnica, 1300-477 Lisboa, Portugal; 5 Centre for the Research and Technology of Agro-Environmental and Biological Sciences, CITAB, Inov4Agro, University of Trás-os-Montes and Alto Douro, 5000-801 Vila Real, Portugal; 6 School of Medicine and Biomedical Sciences (ICBAS), University of Porto, R. de Jorge Viterbo Ferreira 228, 4050-313 Porto, Portugal; 7 Newborn Screening, Metabolism and Genetics Unit, Department of Human Genetics, National Institute of Health Doutor Ricardo Jorge, INSA I.P., Rua Alexandre Herculano, 321, 4000-055 Porto, Portugal; paulo.gaspar@insa.min-saude.pt

**Keywords:** stem cells, iPSCs, cellular model, ML II, lysosomal storage disorder, LSD

## Abstract

Mucolipidosis type II (ML II) is a rare and fatal disease of acid hydrolase trafficking. It is caused by pathogenic variants in the *GNPTAB* gene, leading to the absence of GlcNAc-1-phosphotransferase activity, an enzyme that catalyzes the first step in the formation of the mannose 6-phosphate (M6P) tag, essential for the trafficking of most lysosomal hydrolases. Without M6P, these do not reach the lysosome, which accumulates undegraded substrates. The lack of samples and adequate disease models limits the investigation into the pathophysiological mechanisms of the disease and potential therapies. Here, we report the generation and characterization of an ML II induced pluripotent stem cell (iPSC) line carrying the most frequent ML II pathogenic variant [NM_024312.5(GNPTAB):c.3503_3504del (p.Leu1168fs)]. Skin fibroblasts were successfully reprogrammed into iPSCs that express pluripotency markers, maintain a normal karyotype, and can differentiate into the three germ layers. Furthermore, ML II iPSCs showed a phenotype comparable to that of the somatic cells that originated them in terms of key ML II hallmarks: lower enzymatic activity of M6P-dependent hydrolases inside the cells but higher in conditioned media, and no differences in an M6P-independent hydrolase and accumulation of free cholesterol. Thus, ML II iPSCs constitute a novel model for ML II disease, with the inherent iPSC potential to become a valuable model for future studies on the pathogenic mechanisms and testing potential therapeutic approaches.

## 1. Introduction

Mucolipidosis type II (ML II) or inclusion cell (I-cell) disease (OMIM 252500) is a rare autosomal recessive lysosomal storage disease (LSD) affecting hydrolase trafficking, characterized as a multi-systemic disease with prenatal or neonatal onset and a fatal outcome in early childhood [1]. ML II patients present severe skeletal abnormalities, facial dysmorphia, global developmental delay, cardiorespiratory dysfunction, and hepatosplenomegaly, among other complications [1]. ML II has an incidence per 1,000,000 live births of 2.5–10 cases worldwide, and 1.6 cases in Portugal [2].

ML II is caused by loss of N-acetylglucosamine (GlcNAc)-1-phosphotransferase (here abbreviated as GlcNAc-PTS), a key enzyme to generate the mannose 6-phosphate (M6P) recognition marker on more than 70 lysosomal hydrolases. M6P is essential for the proper recognition of newly synthesized hydrolases by M6P receptors and their subsequent targeting to the lysosome. Without M6P, these enzymes become secreted, and the lysosomes do not function properly due to the accumulation of non-degraded macromolecules, which impair cellular function, ultimately triggering a severe multisystemic pathology [2].

GlcNAc-PTS is a Golgi-resident transmembrane enzyme composed of three subunits (α2β2γ2) encoded by two genes: *GNPTAB,* which encodes the α- and β-subunits, and *GNPTG*, which encodes the γ-subunit [2]. ML II is exclusively caused by pathogenic variants in *GNPTAB* that affect the enzymatic complex in such a way that its function is abolished [2]. The most frequent one is the frameshift c.3503_3504del [NM_024312.5(GNPTAB):c.3503_3504del (p.Leu1168fs)] [1]. The TC dinucleotide deletion on exon 19 of *GNPTAB* disrupts the reading frame, preventing the production of an active enzyme [3]. Remarkably, this variant accounts for 45% of mutant alleles in Portugal and Brazil, 51% in Italy, and 50% among the Arab-Muslim community, and is due to a unique founder molecular lesion that arose ~2000 years ago [4].

Currently, there is no cure or disease-modifying treatment available for ML II. One of the challenges in moving forward with the research of ML II disease and the development of therapies for it is the lack of disease-relevant cellular models. The *in vitro* models available are mostly patient-derived fibroblasts, which, although having the specific genetic heritage, are primary cell cultures with a limited number of passages and which grow very poorly. In addition, ML II is a very rare and rapidly evolving disease, for which it is difficult to obtain patient-derived samples for research purposes.

The generation of induced pluripotent stem cells (iPSCs) has enabled the reprogramming of easily accessible somatic patient cells, such as fibroblasts, into a pluripotent state [5]. Because iPSCs can potentially give rise to any disease-relevant cell type, this technology provides a powerful platform for developing suitable disease models by subsequent differentiation of iPSCs into specific cell types of interest [6]. In addition, still in their pluripotent state, iPSCs also allow for high-throughput drug screening within the patient’s own genetic background and cellular environment, while also bypassing ethical concerns. In fact, several studies have recently been developed using patient-specific iPSCs for modeling other LSDs [7,8,9,10,11,12,13,14]. However, to the best of our knowledge, there are no records of iPSCs from ML II.

In this study, we report the generation and characterization of an ML II patient-specific iPSC line carrying the most frequent pathogenic variant in *GNPTAB*, the c.3503_3504del in homozygosity. This line fulfilled all the requirements of an iPSC line, so it was already registered at the Human Pluripotent Stem Cell Registry under the hPSCreg name INSAi003-A. Furthermore, we report here that ML II iPSCs present the hallmark ML II biomarkers used to diagnose the disease by comparing them with a wild-type (WT) iPSC line and with ML II fibroblasts. Overall, we aimed to demonstrate the usefulness of ML II iPSCs, still in their pluripotent state, as a cellular model of ML II.

## 2. Results

### 2.1. Human ML II Fibroblasts Were Reprogrammed into iPSC-like Colonies

**Figure 1:**
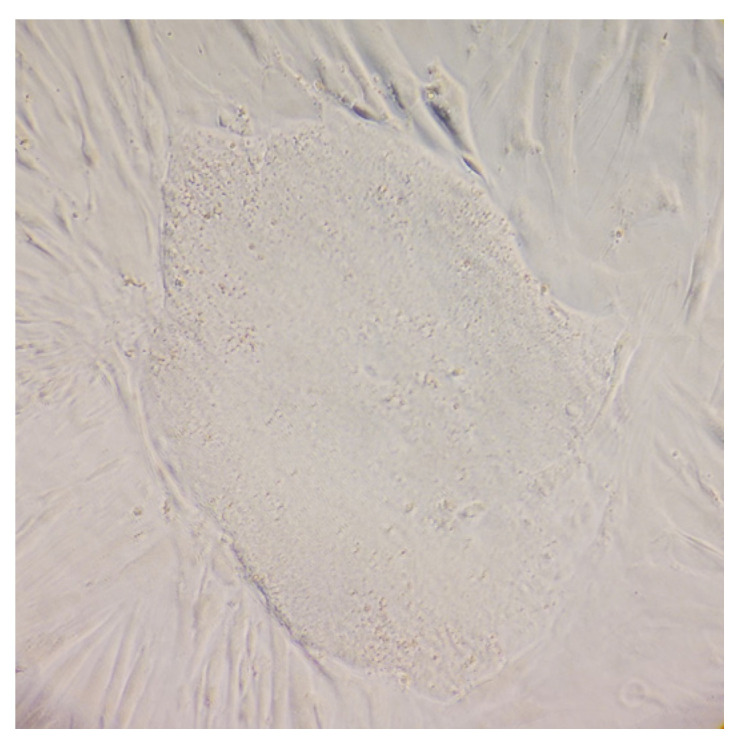
ML II fibroblast culture after reprogramming, showing a colony with clear edges and a typical pluripotent stem cell morphology. Cells were observed using a Leica DMIL inverted contrast microscope (Leica Microsystems, Wetzlar, Germany) with 100× magnification.

To generate iPSCs from ML II patient’s fibroblasts, obtained from Istituto Giannina Gaslini, we used a commercial kit that contains a mixture of five vectors that can reprogram somatic cells to iPSCs without integration. Three episomal vectors deliver the five reprogramming factors (*Oct4*, *Sox2*, *Klf4*, *L-Myc*, and *Lin28*), and the other two vectors express *mp53DD* (a dominant negative mutation of *p53*) and *EBNA1*, which together improve the reprogramming efficiency of the system. After transfection, the fibroblasts experienced morphological changes, from the initial spindle shape to a polygonal or epithelial-like cell morphology. By day 28 after transfection, the first of two colonies with clear edges appeared, which were later picked and expanded, showing a typical pluripotent stem cell morphology (Figure 1).

Although the reprogramming efficiency of the ML II fibroblasts was very low, this was already expected due to the metabolic defects underlying ML II that could potentially hinder the generation of iPSCs. Nonetheless, a clone was chosen after passage (P) 12 to be fully characterized.

### 2.2. iPSC Pluripotency Characterization

#### 2.2.1. iPSCs Were Free from Reprogramming Vectors and Expressed the Key Pluripotency Markers Endogenously

**Figure 2:**
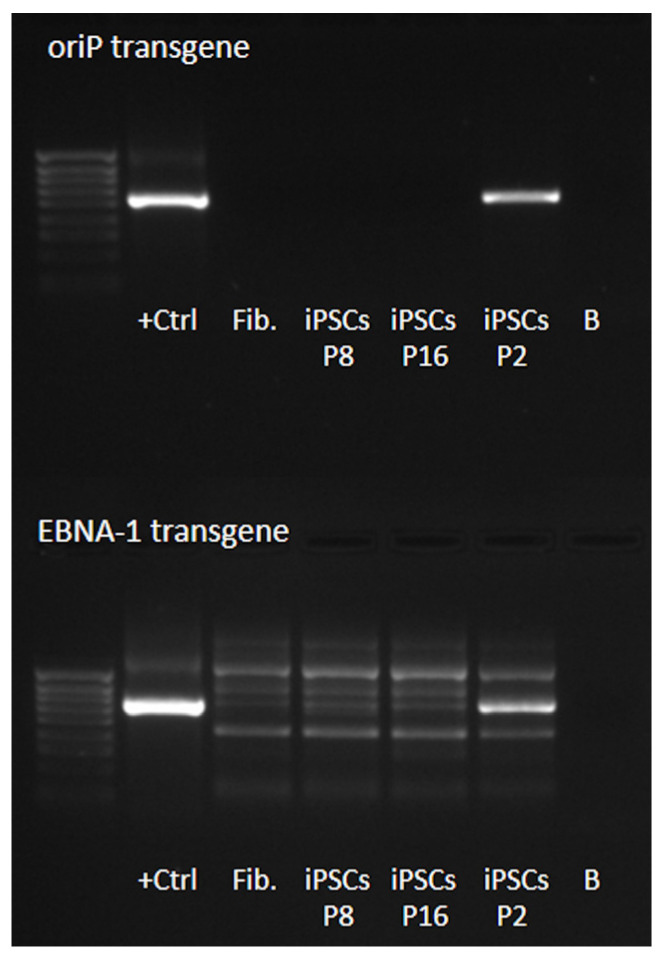
PCR amplification of *oriP* and *EBNA-1* transgenes for detection of Epi5™ reprogramming vectors in ML II iPSCs in passages (P) 2, 8, and 16. As a negative control, ML II fibroblasts (Fib., lane 3) were included. Positive controls (+Ctrl) are the reprogramming vectors containing *oriP* and *EBNA-1* genes. The 100 bp DNA ladder, stained with 6x TriTrack Loading Dye, was used as a molecular weight DNA ladder (lane 1). +Ctrl—positive control; Fib—ML II fibroblasts; iPSCs—ML II iPSCs; B—blank (no DNA template).

According to the manufacturer of the reprogramming kit, “approximately 5% of the episomal vectors are lost each cell cycle due to silencing of the viral promoter driving *EBNA-1* expression and defects in vector synthesis and partitioning”. So, we evaluated the absence of episomal vectors in ML II iPSCs by PCR amplification of the *oriP* and *EBNA-1* transgenes at three different passages (P2, P8, and P16), using ML II fibroblasts as a negative control, and the reprogramming vectors containing *oriP* and *EBNA-1* genes as positive controls. As expected, reprogramming vectors were still detected in P2, but by P8, the iPSCs were already free of vectors (Figure 2).

**Figure 3:**
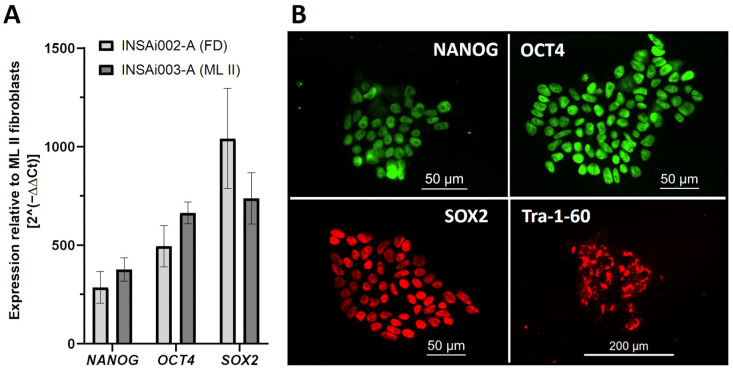
Endogenous expression of pluripotency markers in ML II iPSCs. (**A**) Expression levels of the pluripotency genes *NANOG*, *OCT4,* and *SOX2* in ML II iPSCs relative to the parental ML II fibroblasts, measured by RT-qPCR. The results were normalized to the housekeeping gene *GAPDH*, and expression levels were calculated relative to the expression in the fibroblasts by the 2^−∆∆Ct^ method. As a positive control, an FD iPSC line previously published [INSAi002-A (FD)] [16] was used. Graphs were constructed in GraphPad Prism 8.0.2 and represent the mean and standard deviation of three independent experiments. (**B**) Immunofluorescence in ML II iPSCs showed positive staining for the nuclear pluripotency markers NANOG, OCT4, and SOX2, as well as for the surface pluripotency marker TRA-1-60. Cells were analyzed on a DM400 M fluorescence microscope (Leica), and images were captured and processed in Leica Application Suite v.3.7.0 software.

Knowing that the iPSCs were already free of vectors, we evaluated the endogenous expression of the key pluripotency markers NANOG, OCT4, and SOX2, which play a role in the maintenance of an undifferentiated state and self-renewal ability while repressing genes involved in differentiation pathways, being widely used as iPSC markers [15]. Gene expression analysis by reverse transcription real-time PCR (RT-qPCR) of *NANOG*, *OCT4*, and *SOX2* genes showed a respective increase of 377 ± 60, 664 ± 55, and 737 ± 130 times in ML II iPSCs compared to the patient’s native fibroblasts (Figure 3A). In addition, the expression levels in ML II iPSCs were comparable to an iPSC line from Fabry Disease [INSAi002-A (FD)] previously described by our group [16]. Expression of these transcription factors was also confirmed in ML II iPSCs at the protein level by immunofluorescence, which showed positive staining for these nuclear pluripotency markers, as well as for the pluripotency surface marker TRA-1-60 (Figure 3B). Altogether, these results confirm the pluripotency of the ML II iPSCs.

#### 2.2.2. ML II iPSCs Are Capable of Differentiating into the Three Embryonic Germ Layers

**Figure 4:**
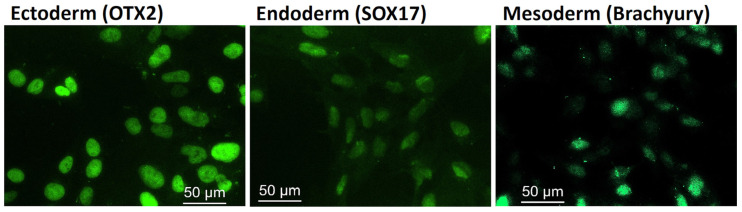
Immunofluorescence showing positive staining for the three germ layer markers OTX2, SOX17, and Brachyury after ML II iPSCs differentiation into ectoderm, endoderm, and mesoderm, respectively. Cells were analyzed on a DM400 M fluorescence microscope (Leica), and images were captured and processed in the Leica Application Suite v.3.7.0 software.

In order to further validate the ML II iPSC pluripotency, we promoted *in vitro* trilineage differentiation into the ectoderm, mesoderm, and endoderm by directed differentiation using a commercial kit with defined media and supplements. Immunofluorescence showed positive staining for specific markers: OTX2 for ectoderm, SOX17 for endoderm, and Brachyury for mesoderm (Figure 4). These results, together with the previously described increased expression of the pluripotency markers, confirmed the functional pluripotency of the ML II iPSC line and capacity to be differentiated into the three germ layers, which in the future will enable differentiation into the most relevant cell types for ML II.

### 2.3. Genetic Characterization Confirmed That ML II iPSCs Have a Normal Karyotype, Were Derived from ML II Patient’s Fibroblasts, and Harbor the Most Frequent Pathogenic Variant

**Figure 5:**
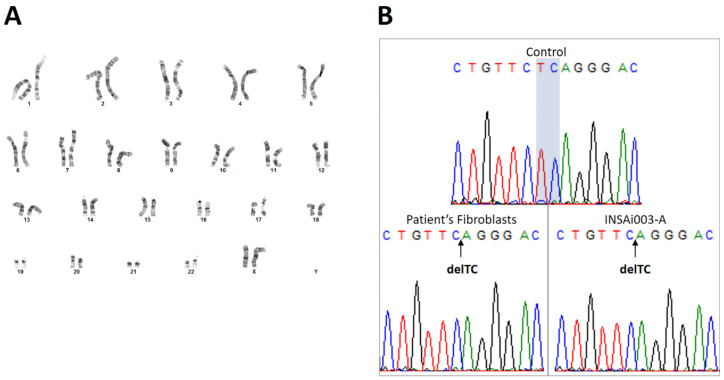
Genetic characterization of the ML II iPSCs. (**A**) G-banded standard karyotype showing a normal female karyotype (46, XX). (**B**) Sanger sequencing chromatograms showing a TC deletion (delTC) both in ML II patient’s fibroblasts and in ML II iPSCs INSAi003-A, which corresponds to the pathogenic variant NM_024312.5(GNPTAB):c.3503_3504del (p.Leu1168fs). The TC nucleotides are present (highlighted) in the WT control sample. The arrows indicate the position in the exon 19 sequence where the TC deletion occurs.

The reprogramming of somatic cells into iPSCs and their expansion up to high passages can increase genetic instability and result in chromosomal abnormalities [17]. To check the karyotype, G-banding analysis of the ML II iPSCs at P14 indicated a normal female karyotype (46, XX) without structural chromosomal abnormalities (Figure 5A).

In order to confirm that the ML II iPSCs carried the c.3503_3504del pathogenic variant in the *GNPTAB* gene like the original ML II fibroblasts, Sanger sequencing of a PCR product spanning exon 19 of *GNPTAB* and its intronic flanking regions was performed on genomic DNA (gDNA). gDNA from WT adult human dermal fibroblasts (HDFa) was also included as a control. Comparing the chromatograms from the three samples, a TC deletion can be observed both in the ML II fibroblasts as well as in the ML II iPSCs chromatogram, while the nucleotides TC are present in the WT control (Figure 5B). These results confirmed the presence of the pathogenic variant NM_024312.5(GNPTAB):c.3503_3504del (p.Leu1168fs) in ML II iPSCs.

Finally, in addition to the Sanger sequencing that allowed us to confirm the molecular defect underlying ML II in this newly generated iPSC line, to ensure the identity and origin of the patient-derived iPSCs and that no cross-contamination occurred, short tandem repeat (STR) analysis was performed by comparing the STR profile of our ML II iPSCs with that of the parental ML II fibroblasts. A 100% match was obtained between iPSCs and fibroblast STRs (Table 1), which proved that the samples are related and are from the same donor.

With the complete genotypic characterization of the ML II iPSCs, we proceeded to phenotypic characterization to understand whether this cell line can recapitulate the ML II phenotype and thus constitute a good model of the disease.

### 2.4. Enzymatic Activity of M6P-Dependent Hydrolases Is Impaired in ML II iPSCs as in ML II Fibroblasts

Unlike most LSDs that are diagnosed by direct measurement of their underlying enzyme deficiency, ML II is diagnosed through an indirect measurement of the activity of M6P-dependent enzymes. The absence of GlcNAc-PTS prevents the formation of the M6P tag and consequently the proper targeting of the lysosomal enzymes, which instead of being transported to the lysosome are secreted from the cell. The ML II diagnostics is implemented and routinely performed at our affiliated routine lab, “Newborn Screening, Metabolism and Genetics Unit of INSA”, and validated by external entities. The methodology is based on quantifying the activity of specific lysosomal enzymes that are marked with M6P, namely alpha- and beta-galactosidase, hexosaminidase A, and total hexosaminidase, in order to indirectly assess whether GlcNAc-PTS is active or not. As a control, the activity of beta-glucosidase, a lysosomal enzyme also known as beta-glucocerebrosidase, was measured. This enzyme follows a M6P-independent trafficking pathway and is delivered to lysosomes via the lysosomal integral membrane protein-2 (LIMP-2), ensuring comparable activity levels in both WT and ML II cells.

**Figure 6:**
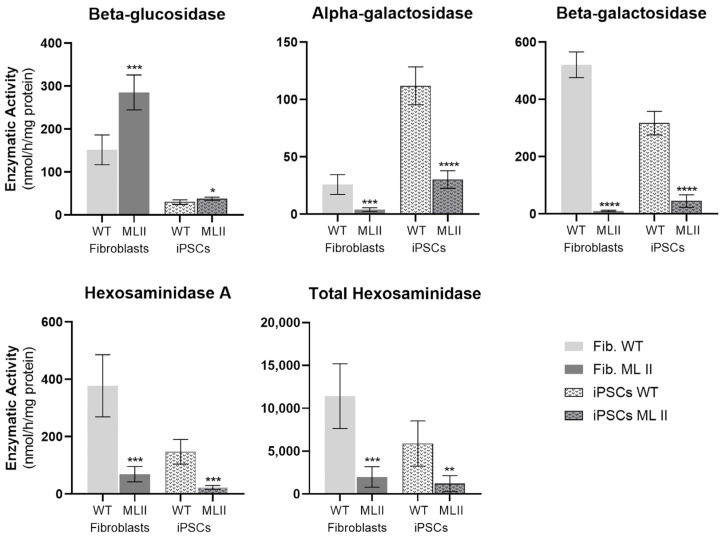
Enzymatic activities of specific hydrolases in ML II and WT fibroblasts and iPSCs. Graphics were constructed in GraphPad Prism 8.0.2. and represent the mean and standard deviation of five independent experiments. Data were analyzed by the Student’s *t*-test between the respective cell types (ML II fibroblasts vs. WT fibroblasts and ML II iPSCs vs. WT iPSCs). * *p* ≤ 0.05, ** *p* ≤ 0.01, *** *p* ≤ 0.001 and **** *p* ≤ 0.0001 vs. WT.

Therefore, we used the same methodology to assess the enzymatic activity patterns both in fibroblasts and iPSCs from ML II and WT samples. Overall, we found differences in the absolute values of enzymatic activities between fibroblasts and iPSCs (Figure 6). However, and most importantly, when comparing the WT with the ML II cells, there is a significant decrease in the activity of the M6P-dependent hydrolases in the ML II cells compared to WT cells (Figure 6 and Table 2).

Regarding the M6P-independent enzyme beta-glucosidase, we observed a slight increase (1.3 ± 0.1 fold) in ML II iPSCs compared to WT iPSCs, and this increase is more evident (1.9 ± 0.3 fold) in ML II fibroblasts compared to the respective WT fibroblasts. This is probably a compensatory mechanism, as not all the M6P-dependent enzymes are functional, and their substrates are probably being accumulated in the lysosomes. The fact that this difference is more pronounced in fibroblasts may be related to their lower division rate compared to iPSCs, which could lead to a greater overload of the cell in terms of undegraded substrates accumulated. Despite the differences in the absolute activity values between fibroblasts and iPSCs, when calculating the ratios between the activities in the respective ML II and WT cells, we found that they are comparable, with the exception of beta-galactosidase, which is particularly low in ML II fibroblasts (Table 2). Overall, these results show that the biochemical defects resulting from the c.3503_3504del pathogenic variant are also present in ML II iPSCs.

**Figure 7:**
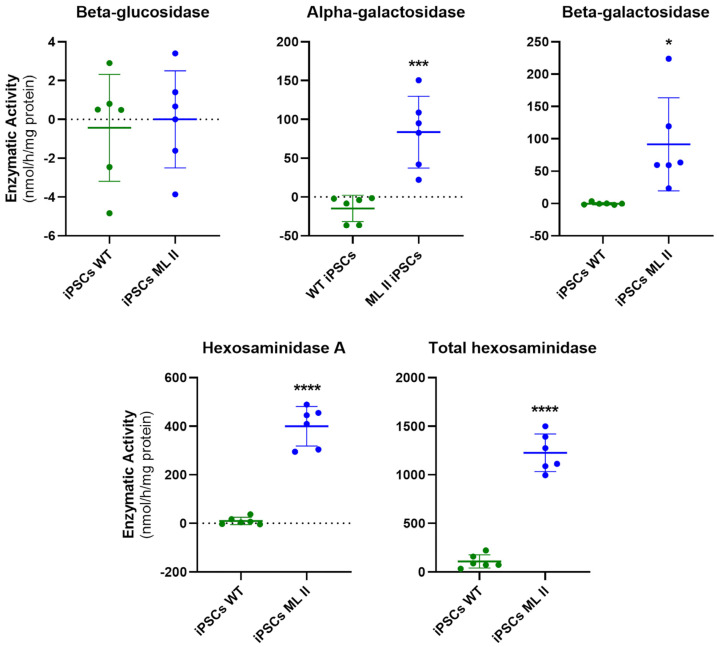
Enzymatic activities of specific hydrolases in conditioned culture media of ML II and WT iPSCs. Graphics were constructed in GraphPad Prism 8.0.2. and represent individual values, as well as the mean and standard deviation of six independent samples. Data were analyzed by the Student’s *t*-test (ML II iPSCs vs. WT iPSCs). * *p* ≤ 0.05, *** *p* ≤ 0.001 and **** *p* ≤ 0.0001 vs. WT.

Aiming to further reinforce these observations, we also measured the enzymatic activities in conditioned cell culture media to confirm increased lysosomal enzyme secretion of M6P-dependent hydrolases. For diagnostic purposes, confirmation is performed by measurement of enzymatic activities in plasma, and results are analyzed concomitantly with those obtained in leukocytes. Thus, we mimicked that rationale for cell cultures. Still, in media, it is not possible to normalize the results obtained, and they will depend on both the confluency and viability of the cells, among other factors such as the composition and pH of the media [18]. However, we know from the clinical diagnosis that the enzymatic activities in plasma under normal conditions (WT) are zero or close to zero because the cells do not secrete these enzymes. Based on this principle, we performed the measurements in conditioned media that were more confluent in WT than in ML II cells, since they would always be close to zero independently of the cell number. By doing so, we ensured that activities higher than zero in ML II compared to WT would not be due to experimental variability related to a higher number of ML II cells. Also, taking into account there were several confounding effects that could hinder proper activity measurements in conditioned media collected from fibroblasts (e.g., the presence of a high percentage of fetal bovine serum, essential for ML II fibroblasts to grow and that could not be removed by buffer exchange), we performed this confirmatory analysis only in the conditioned media of ML II and WT iPSCs. Again, we observed significant differences in the enzymatic activity levels between ML II and WT iPSCs for all assessed M6P-dependent hydrolases, and no significant differences for beta-glucosidase (Figure 7), which showed activities close to zero, confirming it was not secreted by either cell line. As expected, the activities of the M6P-dependent hydrolases were increased in ML II conditioned media, even using a lower cell number, and close to zero in WT, confirming their abnormal secretion by ML II cells. Of note, as mentioned above, this method depends on several factors, which justify the variability observed between experiments, and we showed this by representing the individual values in scatter plots. However, for all M6P-dependent hydrolases, no individual ML II determination falls within the WT range, and vice versa, allowing for a clear distinction and statistically significant differences between ML II and WT in all instances.

### 2.5. Western Blot Results Further Support Abnormal Trafficking of a M6P-Dependent Enzyme, Alpha-Galactosidase

**Figure 8:**
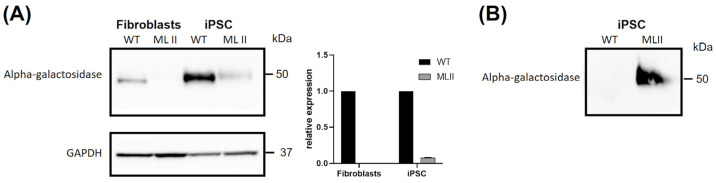
Western blotting for alpha-galactosidase. (**A**) Cellular extracts from WT and ML II fibroblasts and iPSCs. GAPDH was used as a loading control, and densitometric analyses of alpha-galactosidase levels were normalized for GAPDH. Two independent experiments were performed. (**B**) Conditioned media from WT and ML II iPSCs analyzed for alpha-galactosidase.

As already mentioned in the previous section, alpha-galactosidase, the enzyme deficient in Fabry Disease, is one of the many lysosomal hydrolases whose intracellular trafficking depends upon the addition of an M6P marker. Taking advantage of the availability of an optimized Western blot protocol for its detection in-house, we assessed the levels of this protein in cellular extracts, as well as in conditioned media (Figure 8). We followed the same rationale described for enzyme activity assays: intracellular levels were assessed for both fibroblasts and iPSCs, while extracellular levels were only evaluated for iPSCs. As anticipated, protein levels of alpha-galactosidase in cellular extracts were significantly higher in WT than in ML II cells, both for fibroblasts and iPSCs (Figure 8A). Contrarily, a clear shift in this expression pattern was observed in conditioned media from iPSCs. No alpha-galactosidase was detected in the media of WT, while high levels were detected in the media of ML II iPSCs (Figure 8B). Altogether, these results further support that there is a significantly increased secretion of this M6P-dependent enzyme towards the extracellular space.

### 2.6. Filipin Staining Suggests Accumulation of Free Cholesterol in ML II iPSCs

**Figure 9:**
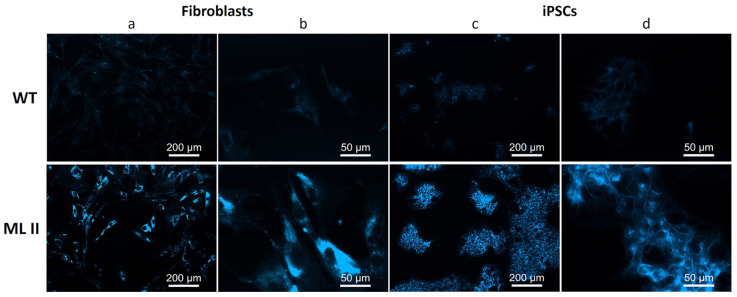
Filipin staining in WT and ML II fibroblasts (**a**,**b**) and WT and ML II iPSCs (**c**,**d**). Cells were analyzed on a DM400 M fluorescence microscope (Leica), and images were captured and processed in Leica Application Suite v.3.7.0 software.

The fluorescent molecule Filipin is used for the localization and quantification of free cholesterol in cells, as it binds to it but not to esterified cholesterol. It is particularly useful for the study of Niemann–Pick type C disease (NPC), an LSD characterized by cholesterol accumulation within lysosomes [19]. However, since one of the defective proteins in NPC is also delivered to lysosomes via the M6P pathway, cholesterol is also stored in ML II fibroblasts [20]. Therefore, Filipin staining was used to analyze the cellular phenotype of ML II iPSCs in terms of free cholesterol accumulation. As a negative control, a WT iPSC line was used, which had been previously established in our group by reprogramming control HDFa cells. ML II fibroblasts were used as a positive control. Comparing WT with ML II cells, either fibroblasts or iPSCs, it was possible to observe a significant increase in the fluorescence staining in ML II cells (Figure 9). Of note, the settings used for image capturing were chosen so that the fluorescence in the ML II cells presented minimal saturation, which led to almost imperceptible labeling in the WT cells. These results demonstrate the accumulation of free cholesterol in ML II iPSCs, as in the ML II positive control fibroblasts.

### 2.7. ML II iPSCs Have a Higher Proliferation Rate than ML II Fibroblasts

**Figure 10:**
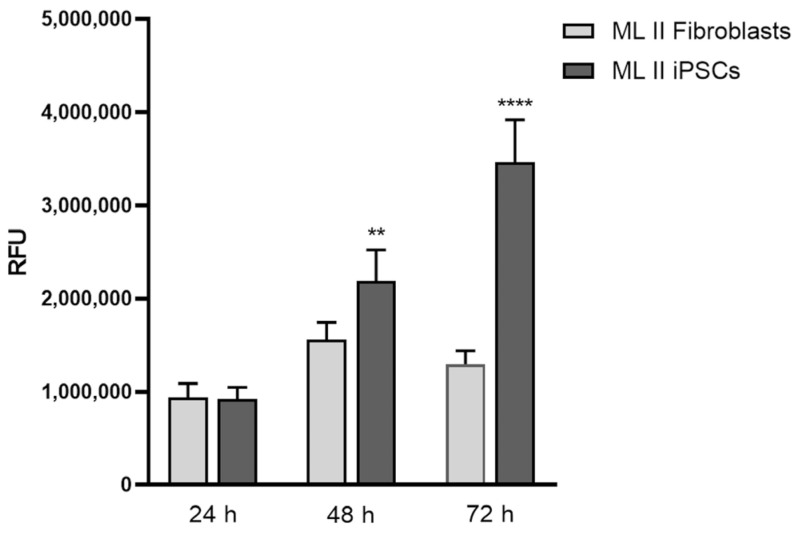
Cell proliferation was assessed by Alamar Blue assay on ML II fibroblasts and iPSCs after 24 h, 48 h, and 72 h of culture. Data were analyzed by the Student’s *t*-test (ML II iPSCs vs. ML II fibroblasts). ** *p* ≤ 0.01 and **** *p* ≤ 0.0001 vs. ML II fibroblasts.

Alamar Blue assay functions as an indicator of cell health, detecting metabolically active cells. It uses resazurin, a non-toxic, blue, and non-fluorescent compound that metabolically active cells reduce to resorufin, a pink, fluorescent, and highly water-soluble product [21]. Thus, it enables the spectrophotometric measurement of cellular viability and proliferation. In this work, we compared the cell proliferation of ML II fibroblasts with ML II iPSCs after seeding the same number of cells. It was possible to confirm that after 48 h, the number of iPSCs is already significantly higher than that of fibroblasts, and this difference became even more pronounced after 72 h (Figure 10). Furthermore, iPSCs showed linear growth over time, whereas fibroblasts divided more slowly and appeared to have reached a plateau after 72 h. Interestingly, this result is in agreement with our empirical observations after extracting nucleic acids from both lines, in which we consistently obtained a lower quantity of both DNA and RNA in fibroblasts than in iPSCs, after having seeded the same number of cells and having incubated them for the same period of time. These results were somehow expected, as iPSCs are pluripotent, thus having virtually the potential to divide indefinitely, although their longevity may depend on multiple factors, including culture conditions, genetic integrity, and epigenetic stability [22]. On the other hand, fibroblasts are primary cells with a limited passage number. So, we showed here one of the advantages of iPSCs compared to fibroblasts as ML II models, which is to obtain a greater number of cells in less time and consequently a greater quantity of biological sample, this being useful, for example, for high-throughput assays.

## 3. Discussion

Historically, fibroblasts have been the most common cellular models for LSDs in general, and, in particular, for ML II (or I-cell) disease. They have proven very useful not only to uncover the mechanisms of the disease, but also for diagnostic purposes [20,23]. In fact, the only human cellular models for ML II available so far are the patient-derived fibroblasts. More recently, Kose et al. [24] reported the characterization of mesenchymal stem cells of ML II but did not specify the pathogenic variant associated. Patient cells have the advantage of having the same genetic background and allow for close recapitulation of transcriptomic and proteomic profiles and thus proximity to physiological function.

However, patients’ samples are scarce because ML II is a very rare disease, with patients presenting a severe phenotype and mostly dying within the first decade of life [1,2]. This also impairs the development of therapeutic approaches for this disease, which still has no cure. Therefore, it would be advantageous to have disease-relevant cell types derived from patients that accurately mimic the disease phenotype, allow for high-throughput drug screenings, and ideally represent the most affected cells. With the iPSC technology, it is possible to reprogram somatic cells from patients to pluripotency and use either the iPSCs in their pluripotent state or differentiate them into the most important cell types. They have certain advantages over other cell models, related to their versatility, such as having an almost unlimited expansion capacity, fast cellular division, being amenable to genetic engineering, and being able to be differentiated into most types of somatic cells [6,25].

In this sense, here we report, for the first time, the generation and characterization of an iPSC line derived from fibroblasts of an ML II patient homozygous for the most frequent pathogenic variant, the c.3503_3504del [NM_024312.5(GNPTAB):c.3503_3504del (p.Leu1168fs)]. Furthermore, we demonstrated that the ML II iPSC line presents the characteristic ML II phenotype previously described for ML II fibroblasts [20], namely, accumulation of free cholesterol and decreased activity of M6P-dependent hydrolases [20,26]. Thus, to the best of our knowledge, we established the first iPSC model of ML II.

These cells were reprogrammed from fibroblasts using a commercial kit that we had already used to generate iPSCs from a FD patient [16]. It delivers the pluripotency factors through non-integrative episomal vectors, which have a low risk for insertional mutagenesis. Indeed, the genetic characterization showed no chromosomal alterations. For the characterization of this iPSC line, both genetically and in terms of pluripotency, we followed the mandatory guidelines of the Human Pluripotent Stem Cell Registry [27] and, as the cell line fulfilled all the criteria, they were already registered under the name INSAi003-A “https://hpscreg.eu/cell-line/INSAi003-A (accessed on 14 February 2025)”. Overall, we showed that this ML II iPSC line has a normal karyotype and disease-causing pathogenic variant, and the pluripotency properties that make it capable of differentiating into the three germ layers. These properties are fundamental to enable its subsequent differentiation into various cell types for future studies.

In addition to assessing their pluripotency potential, we aimed to evaluate their phenotype in order to validate their usefulness as a cellular model for ML II. To do so, and as fibroblasts are a patient-derived and well-established cellular model for ML II, we included the ML II fibroblasts as a positive control and WT cells, either fibroblasts or iPSCs, as negative controls. In general, fibroblasts obtained by skin biopsy of LSD patients display a major disease hallmark for this particular group of disorders: the storage of un- or partially degraded substrates [23]. Yet, unlike other LSDs that result from individual deficiencies of highly expressed lysosomal enzymes, ML II is the result of defects in a Golgi-resident transmembrane enzyme whose expression levels are naturally low. This means that some of the studies/techniques, which are usually easy to perform in LSD patient-derived fibroblasts, are actually difficult in ML II cells. Amongst those techniques are Western blots and immunofluorescence co-localization studies. Indeed, while for the vast majority of lysosomal enzymes it is feasible to detect endogenous levels of expression with various techniques, most methods fail to detect WT levels of GlcNAc-PTS. To overcome this limitation, some studies have used cellular models overexpressing the human *GNPTAB* sequence, either the WT or ML II-causative variants [28,29]. However, these are still artificial systems. So, unlike most LSDs, ML II is not diagnosed by direct measurement of its underlying enzyme deficiency. Instead, at our affiliated routine lab, “Newborn Screening, Metabolism and Genetics Unit”, as in most diagnostic labs worldwide, it relies on an indirect assay to detect elevated plasma activity of lysosomal M6P-dependent hydrolases and/or their decreased activity in fibroblasts. This is followed by the molecular screening of the *GNPTAB* gene to confirm the diagnosis.

In this work, we measured the enzymatic activity of four M6P-dependent and one M6P-independent hydrolases. As expected, we found a significant decrease in the activity of the M6P-dependent hydrolases, both in ML II fibroblasts and iPSCs, meaning that the GlcNAc-PTS is not active. In contrast, the M6P-independent lysosomal hydrolase beta-glucosidase showed a small increase in ML II cells, which was more pronounced in fibroblasts than in iPSCs, probably because these proliferate much faster and have a higher turnover than fibroblasts. This small increase in beta-glucosidase activity may represent a compensatory mechanism as the cells are overloaded with undegraded substrates. Indeed, an increase in the activity of this enzyme had already been reported by Boonen et al. [30] in different tissues of a *gnptab* knockout mouse model, and by Otomo et al. [20] in human ML II fibroblasts. Importantly, we have also measured the activity of the same enzymes in conditioned culture media collected from both WT and ML II iPSCs. And again, the results we obtained were in agreement with our expectations: enzyme activity levels were similar for the M6P-independent lysosomal hydrolase beta-glucosidase, whilst significantly higher in ML II than in WT conditioned media for all the other tested enzymes. Overall, these parallel and concomitant observations provide clear evidence of the absence of activity of GlcNAc-PTS. It is also worth mentioning that the ratios of activities between ML II and WT iPSCs were comparable to those found between ML II and WT fibroblasts, further supporting how closely this new cellular model mimics the historical gold standard for *in vitro* studies in ML II. This was an important step for the phenotypic characterization of the ML II iPSCs, as this is a hallmark marker of the disease.

Still, to corroborate the results regarding the ability of this model to display a disease-related phenotype in terms of protein expression, we focused our attention on one of the most ubiquitous hydrolases, which has long been known to target the lysosome via the M6P-dependent pathway: alpha-galactosidase. We evaluated its expression levels by Western blot, both in cellular extracts and in cultured media, comparing the results in ML II with WT cells. The results we obtained were a perfect depiction of the consequences of disrupted M6P trafficking: the levels of our target enzyme were significantly lower in ML II cellular extracts compared to WT, and increased in the ML II media, while absent in the WT. Overall, these observations further support the ability this new iPSC line holds to display visible and measurable phenotypes.

To further confirm their usefulness as a cellular model for ML II, we also analyzed the accumulation of free cholesterol by Filipin staining and showed that ML II iPSCs also stored free cholesterol, like ML II fibroblasts. This was expected and had been previously shown by Otomo et al. [20] in ML II fibroblasts, because NPC intracellular cholesterol transporter 2 (NPC2) is a lysosomal enzyme that is targeted to this compartment also via M6P-dependent trafficking [31]. Pathogenic variants in the *NPC2* gene, as well as in the *NPC1* gene, cause NPC disease, which is characterized by the accumulation of unesterified cholesterol and other lipids in endo/lysosomal compartments [32]. Filipin staining is not used for diagnosing ML II, but, despite methodological caveats and variability in the patterns encountered in patients with proven NPC, it is still used in the diagnosis of NPC combined with the molecular methods [19,33]. As Filipin staining is a simple and fast method, it could be useful for future studies using the ML II iPSCs as a cellular model. For example, to test new therapeutic approaches for ML II, Filipin could be useful in a first assessment of the phenotype after exposing the cells to potential treatments.

ML II is also called I-cell disease due to the presence of vacuole-like inclusions, found in fibroblasts and B-cells of ML II patients [34,35]. However, their presence can sometimes be subtle or absent in some cells, such as neurons, skeletal muscle, CD4+ T cells, CD8+ T cells, natural killer cells, monocytes, or neutrophils [35,36]. Therefore, researchers started to realize that their absence does not rule out the disease, especially when supported by clinical symptoms and genetic findings. Interestingly, we observed more vacuole-like inclusions in the ML II fibroblasts compared to WT, but no evident differences were observed between ML II and WT iPSCs (Supplementary File), maybe due to their smaller size and higher turnover. Still, even though detecting those inclusion bodies would be interesting, it would not constitute definitive proof of pathology.

Data on the direct use of undifferentiated iPSCs as disease models is scarce, particularly in the LSD field. Indeed, most authors use the generated patient-derived iPSC lines to further differentiate them into disease-relevant cell types, namely neurons and glia [37,38,39], chondrocytes [40], and cardiomyocytes [41]. Ultimately, organoids may also be produced [14], which by replicating human tissue structure and function, may offer more accurate models to study biological processes, disease progression, and complex multisystem interactions and phenotypes. However, in other fields, namely mitochondrial diseases, aging, or infectious diseases, undifferentiated iPSCs are already being used as disease models *per se*, either to evaluate the pathological phenotypes of the diseases [42,43,44], or for high-throughput screening of therapies [45]. For example, Nguyen et al. [43] have used Port Wine Birthmarks (PWBs) iPSCs as a model to exploit the metabolites associated with the pathological phenotypes of the disease, which is a congenital vascular malformation on the skin. Besides having found significantly affected metabolic pathways in PWB iPSCs compared to normal ones, they validated their most relevant findings by immunohistochemistry in human PWB vasculatures, showing the clinical relevance of this model. In the infectious diseases field, an iPSC-based screening was conducted by Imamura et al. [45] to measure viral infectivity, which resulted in the identification of several FDA-approved drugs able to modulate host cell susceptibility against RNA viruses.

Overall, though, the utility of our newly generated iPSC line as a model for ML II remains somewhat theoretical at this stage, which is a limitation of our work. However, still in their undifferentiated state, we demonstrated the unequivocal measurable disease-related phenotypes in this rapidly dividing, pluripotent cell type, strongly supporting their use as a valuable platform for drug screening and identification of promising therapeutic candidates, in detriment of the ML II fibroblasts that, as we have shown, grow very poorly and have limited passages. This is particularly relevant in the context of drug repositioning studies, for example, where large panels of drugs can be tested against a single disease or disease phenotype, which requires faster, more cost-effective, and less resource-intensive initial drug screenings before full differentiation [46]. The most promising drugs can further be tested in terminally differentiated and fully functional relevant cell types, whose generation and characterization are highly time- and cost-demanding.

Nevertheless, our future plan will be to further differentiate these iPSCs into disease-relevant cell types, namely the extended chondrocyte-osteoblast lineage, the neural cells, and the cardiomyocytes, to unveil the skeletal, neurological, and cardiac phenotypes, respectively. As stated before, however, differentiation into disease-relevant cells and their subsequent functional characterization and disease phenotype assessment is highly time- and resource-demanding. It requires careful planning and investment both in infrastructure and expertise, or collaborations, as these resources may be missing both in resource-constrained academic settings, and in reference laboratories like ours, whose primary function lies between assistance/diagnosis and applied research.

Regardless of these constraints, in the future, we also intend to challenge the utility of both undifferentiated and terminally differentiated ML II iPSCs for preclinical drug development. Indeed, we have a target for those assessments, as studies on a c.3503_3504del mutation-specific therapeutic approach using antisense oligonucleotides (ASOs) are ongoing in our group. Our goal is to induce the skipping of *GNPTAB* exon 19, aiming to circumvent the pathogenic effect of the c.3503_3504del variant, a strategy similar to that successfully applied for Duchenne muscular dystrophy [3]. This approach leads to the production of a smaller but in-frame *GNPTAB* transcript, which we expect will enable the synthesis of an α/β-subunit precursor polypeptide, aimed at partially rescuing enzymatic activity to ameliorate the disease-associated phenotype [3]. Despite exploratory, the effectiveness of specific ASOs was confirmed in patients’ fibroblasts and paved the way for future studies. The ML II iPSCs will make it possible to extend the studies we have carried out on fibroblasts to other cells more relevant to ML II pathology.

Overall, this newly generated ML II iPSC line displays the major advantages and disadvantages of any iPSC line. On the one hand, it has a fast division rate and constitutes an unlimited supply of patient-specific cells. On the other hand, it displays a pluripotent phenotype and does not represent, *per se*, a realistic model of human disease, as it may fail to recapitulate, in its pluripotent state, complex phenotypes that may be cell-specific. Altogether, though, the advantages of using iPSCs and their derivatives for modeling LSDs, namely in the study of the pathophysiological mechanisms and in drug screening and preclinical evaluation of novel therapies, are well documented [8,12,13,14,39,41,47], but not for ML II. And while it was not possible for us at this stage to perform a phenotypic characterization of this first ML II iPSC line in terms of full differentiation capacity into a disease-relevant cell type, we do believe it is important to report the presence of hallmark and measurable disease phenotypes even in this undifferentiated, pluripotent stage. Now we expect that this ML II iPSC line, and more that may be generated in the future for other pathogenic variants, will significantly boost advancements in research on this rare disease.

## 4. Materials and Methods

### 4.1. Cell Cultures

Skin fibroblasts from an ML II patient, homozygous for the *GNPTAB* frameshift variant c.3503_3404del, were obtained from the “Cell Line and DNA Biobank from Patients Affected by Genetic Diseases” (Istituto Giannina Gaslini, Genova, Italy; member of the Telethon Network of Genetic Biobanks) under the biobank’s guidelines [48]. Human Dermal Fibroblasts, adult (HDFa), were purchased from ThermoFisher Scientific (Waltham, MA, USA) and used here as control WT fibroblasts. We had previously reprogrammed these HDFa to pluripotency, and the generated iPSCs were used here as control WT iPSCs. An iPSC line of Fabry Disease [INSAi002-A (FD)] had been previously generated and described [16] and was used here as a positive control for the pluripotency analysis. Fibroblasts were cultured in DMEM with GlutaMax and 10% FBS (all Gibco, ThermoFisher Scientific, Waltham, MA, USA), and iPSCs were cultured in vitronectin-coated dishes using Essential 8™ Flex Medium (all Gibco, ThermoFisher Scientific), and kept at 37 °C, 5% CO_2_, 20% O_2_.

### 4.2. Reprogramming ML II Fibroblasts into iPSCs

ML II fibroblasts at P6 were cultivated on vitronectin-coated dishes and reprogrammed using the Epi5™ episomal iPSC reprogramming kit with Lipofectamine™ 3000 Transfection Reagent (both Invitrogen, ThermoFisher Scientific, Waltham, MA, USA), according to the manufacturer. Briefly, the kit contains reprogramming vectors that induce the expression of the pluripotency genes *OCT4*, *SOX2*, *LIN28*, *KLF4*, and *L-MYC*, and, for increased reprogramming efficiency, *mp53DD* and *EBNA1*. This kit ensures viral-free and integration-free reprogramming, guaranteeing the loss of episomal vectors at a 5% rate per cell cycle. Colonies exhibiting an iPSC-like appearance were manually isolated and expanded.

### 4.3. Culture and Expansion of iPSCs

iPSCs were cultured under feeder-free conditions, on vitronectin-coated dishes in Essential 8™ Flex Medium (all Gibco, ThermoFisher Scientific, Waltham, MA, USA) at 37 °C, 5% CO_2_, 20% O_2_. Cells were passaged at 1:6–1:8 ratio every 4–5 days with 0.5 mM EDTA in DPBS. Regular testing for Mycoplasma was performed by PCR for the 16S rRNA gene using an in-house protocol with the following primers: 5′-TGCACCATCTGTCACTCTGTTAACCTC-3′ and 5′-ACTCCTACGGGAGGCAGCAGTA-3′.

### 4.4. Detection of Episomal Vectors

DNA was extracted from cellular pellets of ML II iPSCs at P2, P8, and P16 and from ML II fibroblasts (P6) using the QIAamp^®^ DNA Blood Mini Kit (Qiagen, Hilden, Germany). The Epi5™ reprogramming vectors and the Epi5™ P53 and EBNA vectors were diluted in nuclease-free water to final concentrations of 1 µg/µL and 0.25 µg/µL of DNA, respectively, and used as positive controls. Detection of episomal iPSC reprogramming vectors was performed by PCR using the EBNA-1 and oriP primer pairs and conditions recommended in the kit.

### 4.5. Immunostaining of the Pluripotency and Differentiation Markers

Pluripotency of ML II iPSCs was determined with Fluorescent Human ES/iPS Cell Characterization Kit (Merk Milipore, Burlington, MA, USA), and their differentiation into the three germ layers (endoderm, mesoderm and ectoderm) was assessed using the Human Pluripotent Stem Cell Functional Identification Kit (R&D Systems^®^, Minneapolis, MN, USA), according to manufacturer’s instructions. To differentiate into mesoderm, the respective media from the kit was supplemented with 3 µM CHIR99021 (STEMCELL™ Technologies, Vancouver, BC, Canada), a WNT pathway activator that promotes direct mesoderm differentiation. The antibodies used were those included in the kits. The slides were mounted in VECTASHIELD^®^ Antifade Mounting Medium with DAPI (Vector Laboratories, Newark, CA, USA). Cells were analyzed on a DM400 M fluorescence microscope (Leica, Wetzlar, Germany).

### 4.6. Reverse Transcription and Quantitative Real-Time PCR (RT-qPCR)

RNA from ML II iPSCs and from the ML II fibroblasts that originated them, as well as from the iPSC line of FD [INSAi002-A (FD)] that had already been published [16], was extracted with TRIzol™ Reagent (Invitrogen, ThermoScientific, Waltham, MA, USA), followed by reverse transcription from 600 ng of RNA using the NZY M-MuLV First-Strand cDNA Synthesis Kit (NZYTech, Lisboa, Portugal). Expression of the pluripotency genes *NANOG*, *OCT4*, and *SOX2* was determined by qPCR (three independent experiments, with two technical replicates) using the NZYSupreme qPCR Probe Master Mix (NZYTech, Lisboa, Portugal) and TaqMan^®^ Gene Expression assays (Hs02387400_g1, Hs00999632_g1, and Hs01053049_s1, respectively). Reactions were performed in a CFX96 Touch™ Real-Time PCR Detection System and data processed in Bio-Rad CFX^®^ Manager Software 3.1 (both Bio-Rad Laboratories, Hercules, CA, USA). Ct-values were normalized to the housekeeping gene *GAPDH* (Hs02786624_g1) using the standard ΔCt method. Relative expression in iPSCs compared to fibroblasts was calculated (2^−ΔΔCt^) [49] and expressed as mean ± standard deviation.

### 4.7. Genetic Characterization

A fragment of the *GNPTAB* gene was PCR amplified using the primer pair 5′-CCCATAGCTAAAAGGCCATCTACC-3′/5′-GTATACACTCACCCACACACATGC-3′ and analyzed through Sanger sequencing to confirm the c.3503_3504del variant in ML II iPSCs (P12), and compared to both ML II and WT fibroblasts (Gibco, ThermoFisher Scientific, Waltham, MA, USA). For STRs, the AmpFLSTR^®^ Identifier^®^ (Applied Biosystems™, Waltham, MA, USA) was used to analyze DNA from ML II fibroblasts (P5) and iPSCs (P16). For karyotyping, ML II iPSCs were dissociated using 0.5 mM EDTA in DPBS, plated onto T25 flasks, and cultured until 50–60% confluency. Cells were then sent to the Cytogenetics Unit of DGH-INSA for karyotype analysis on G-banded metaphase chromosomes using standard procedures. At least four metaphases were examined per sample.

### 4.8. Measurement of Enzymatic Activity

For the cellular extracts, cellular pellets were homogenized in potassium phosphate buffer (25 mM, pH 6.5) with 0.1% (*v*/*v*) Triton X-100. Extracts were prepared by sonication (40% power, 40% amplitude for 6 s). Protein was quantified with a BCA Kit (ThermoFisher Scientific, Waltham, MA, USA). For the conditioned media, ML II and WT iPSCs at approximately 60% and 80% confluency, respectively, were maintained in Essential 8™ Flex Medium. After 24 h, 2 mL of media was buffer exchanged with DPBS using Amicon^®^ Ultra-15 centrifugal filter units and concentrated to a final volume of 500 µL. Enzymatic assays were performed according to a previously described method by Aerts et al. [50] for beta-glucosidase, Gaspar et al. [51] for alpha-galactosidase, Crippa et al. [52] for beta-glalactosidase, and Ribeiro et al. [53] for both hexosaminidase A and total hexosaminidase. Five and six independent measurements were performed for each enzyme analyzed in cellular extracts and in conditioned media, respectively. Enzymatic activities were normalized for the protein quantified in the extracts. Experimental data were expressed as mean ± standard deviation. Statistical analyses were performed using GraphPad Prism 8.0.2. for Windows (La Jolla, CA, USA). Data were analyzed by Student’s *t*-test, used to compare the means between ML II and WT fibroblasts and between ML II and WT iPSCs. Significance was accepted at a *p* value ˂0.05.

### 4.9. Western Blot for Alpha-Galactosidase

For the Western blot analysis of cellular extracts, between 1.4 and 1.6 × 10^6^ fibroblasts and iPSCs were harvested, homogenized in PBS with phenylmethylsulfonyl fluoride (PMSF), and lysed by three cycles of 5 min freeze–thawing (liquid nitrogen and 37 °C water bath). Protein concentration was determined using the Pierce™ BCA Protein Assay Kit (ThermoFisher Scientific, Waltham, MA, USA) and measured in a VICTOR^®^ Nivo^TM^ Plate Reader spectrophotometer (PerkinElmer, Waltham, MA, USA). For conditioned media, samples were obtained as described in 4.6. Then, 20 ng of total protein from cellular extracts and 10 µL of media samples were loaded on a 4–12% NuPAGE^®^Novex^®^Bis-Tris precast gel (Invitrogen™, ThermoFisher Scientific, Waltham, MA, USA) and ran in a XCell4 SureLock Midi-Cell Electrophoresis System (ThermoFischer Scientific, Waltham, MA, USA) at 200 V and 200 mA. Proteins were then transferred to a nitrocellulose membrane using the standard protocol in a Trans-Blot Turbo Transfer System (Bio-Rad Laboratories, Hercules, CA, USA). Immunodetection of alpha-galactosidase was carried out using the rabbit recombinant monoclonal anti-galactosidase alpha antibody [EP5828(2)] (ab168341, Abcam, Cambridge, UK), followed by the secondary antibody goat anti-rabbit IgG-HRP (sc-2004, Santa Cruz Biotechnology, Dallas, Texas, EUA). Total protein loaded was controlled by incubation with a monoclonal anti-GAPDH (V-18) antibody (sc-20357, Santa Cruz Biotechnology, Dallas, Texas, EUA) and the secondary antibody donkey anti-goat IgG-HRP (sc-2020, Santa Cruz Biotechnology, Dallas, Texas, EUA). The immunoblot was developed using the Amersham ECL Detection Reagent (Cytiva, Wilmington DE, USA) and analyzed in the ChemiDoc XRS+ imaging system (Bio-Rad Laboratories, Hercules, CA, USA). Densitometric analysis was carried out using Image Lab Software (version 6.1, Bio-Rad, Hercules, CA, USA), and alpha-galactosidase protein bands were normalized for GAPDH densities. Two independent experiments were performed.

### 4.10. Filipin Staining

Filipin staining was performed in ML II iPSCs. As controls, ML II and WT fibroblasts, as well as WT iPSCs, were used. Briefly, cells were cultured for four days in Lab-TekII 8-well chamber slide plates (Thermo Scientific™, Waltham, MA, USA), then washed with DPBS, after which they were fixed with 4% PFA in PBS for 15 min. Then, cells were washed twice with DPBS for 5 min, followed by incubation with 50 mM NH_4_Cl, a quenching solution, for 10 min. Cells were washed quickly and incubated for 30 min with 125 µg/mL of Filipin III (Sigma-Alrich, St. Louis, MO, USA) in DPBS (note that Filipin powder was first resuspended at 12,5 mg/mL in DMSO and then diluted in DPBS to the final concentration of 125 µg/mL). Cells were visualized on a Leica Fluorescence Microscope DM4000 M and processed in the Leica Application Suite v.3.7.0 software.

### 4.11. Cell Proliferation Assay with Alamar Blue

On day 1, ML II iPSCs and fibroblasts were detached, respectively, with 0.5 mM EDTA in DPBS and Trypsin-EDTA (0.25%) (both ThermoFisher Scientific, Waltham, MA, USA). Cells were counted with a Neubauer Chamber and seeded on 96-well plates at a density of 4 × 10^3^ cells/well. iPSCs were resuspended in Essential 8™ Flex Medium supplemented with RevitaCell and fibroblasts in DMEM supplemented with 10% FBS (all Gibco, ThermoFisher Scientific, Waltham, MA, USA). On day 0, the medium was changed to Essential 8™ Flex Medium (iPSCs) and DMEM 10% FBS (fibroblasts). After 24, 48, and 72 h of incubation, the medium was removed and 100 µL of fresh medium with 1/10th volume Alamar Blue HS (Invitrogen, ThermoFisher Scientific, Waltham, MA, USA) was added to each well. Cells were incubated for 1 h at 37 °C in the cell culture incubator, followed by fluorescence reading using an excitation wavelength of 570 nm and an emission of 610 nm. The results were averaged over one experiment with 3 plates (1 for each time-point) and 6 replicates per plate. Wells containing only the medium with Alamar Blue, without cells, were included, and the fluorescence measured was used as a background and subtracted.

## 5. Conclusions

Here, we describe the establishment of an ML II iPSC line carrying the most frequent pathogenic variant, the c.3503_3504del [NM_024312.5(GNPTAB):c.3503_3504del (p.Leu1168fs)]. These ML II iPSCs demonstrated full pluripotency capacity and presented the characteristic ML II phenotype, namely decreased activity of M6P-dependent hydrolases and accumulation of free cholesterol.

To the best of our knowledge, this is the first ML II iPSC model to be reported, and we demonstrated here its potential usefulness as a cellular model for ML II to boost future research on this disease.

## Figures and Tables

**Table 1:** Analysis of 15 short tandem repeat (STR) markers and Amelogenin in ML II iPSCs and ML II fibroblasts using the AmpFLSTR Identifiler PCR Amplification kit (Applied Biosystems, Warrington, UK).

		Test Sample ML II iPSCs	Reference Sample ML II Fibroblasts
	Passage Number	16	5
STR Markers	D8S1179	13, 14	13, 14
	D21S11	28, 30	28, 30
	D7S820	10, 13	10, 13
	CSF1PO	12	12
	D3S1358	16, 18	16, 18
	TH01	6, 8	6, 8
	D13S317	11	11
	D16S539	9, 11	9, 11
	D2S1338	22, 25	22, 25
	D19S433	12, 13	12, 13
	vWA	17	17
	TPOX	8	8
	D18S51	14, 16	14, 16
	AMEL	X	X
	D5S818	9, 12	9, 12
	FGA	19, 24	19, 24

**Table 2:** Ratio of the respective enzymatic activities between ML II and WT fibroblasts and ML II and WT iPSCs. Values represent the mean and standard deviation of five independent experiments.

	Ratio Activity in ML II/Activity in WT	
	Fibroblasts	iPSCs
Beta-glucosidase	1.9 ± 0.3	1.3 ± 0.1
Alpha-galactosidase	0.16 ± 0.07	0.27 ± 0.07
Beta-galactosidase	0.02 ± 0.01	0.14 ± 0.07
Hexosaminidase A	0.18 ± 0.07	0.16 ± 0.05
Total hexosaminidase	0.18 ± 0.10	0.21 ± 0.16

## Data Availability

The original contributions presented in this study are included in the article/Supplementary Materials. Further inquiries can be directed to the corresponding author(s).

## Supplementary Materials

The following supporting information can be downloaded at: https://www.mdpi.com/article/10.3390/ijms26083871/s1.
