# Supplementary material for: Establishment of a Human iPSC Line from Mucolipidosis Type II That Expresses the Key Markers of the Disease
Source: Int J Mol Sci. 2025 Apr 19;26(8):3871. doi: 10.3390/ijms26083871 (PMC12027929; doi:10.3390/ijms26083871)
Supplement: Supplementary file 1 [file ijms-26-03871-s001.zip › ijms-3474108-supplementary.pdf]

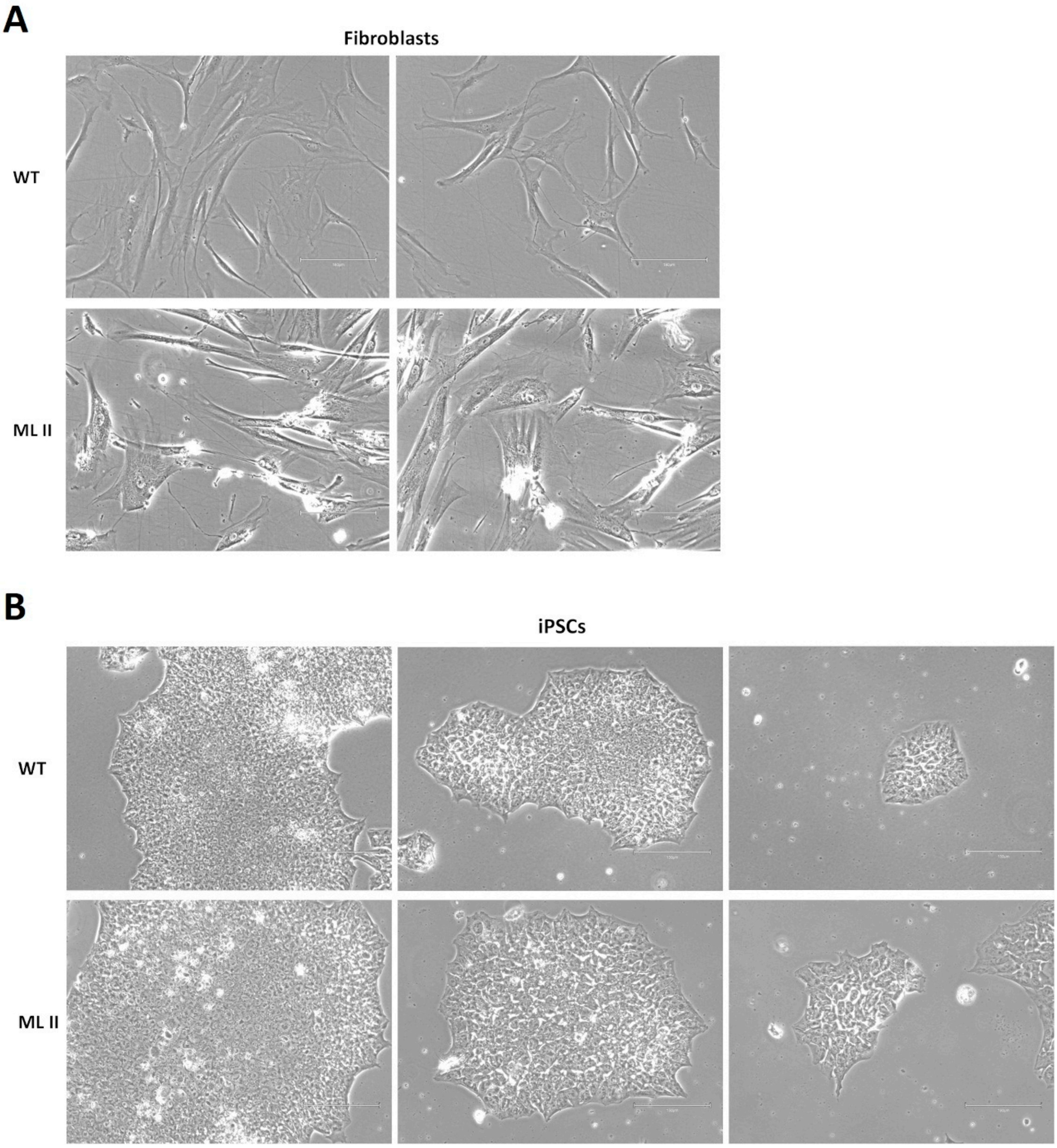

Supplementary Figure: Phase contrast microscopy to assess the presence/absence of inclusion bodies in cultured cells. (A) WT and ML II fibroblasts, showing the presence of vacuole-like inclusions in ML II cells. (B) WT and ML II iPSCs. Here the presence of inclusion bodies is not evident. Cells were visualized using an EVOS M5000 Imaging System (ThermoFisher Scientific) and bars represent 150 µm.
